# A Case of Placenta Prævia, with Faulty Position of the Child

**Published:** 1891-01

**Authors:** T. Enoree Nott


### A CASE OF PLACENTA PRÆVIA, WITH FAULTY POSITION OF THE CHILD.

By T. ENOREE NOTT, Jr.

I was called to see Mrs. J. W. on the 24th of August last; found her nearly seven months pregnant, anæmic, and having occasional uterine contractions, accompanied by slight hemorrhage. Digital examination revealed nothing abnormal. No dilatation of the os having taken place. The ordinary routine was ordered; rest in bed, opium and black haw, under which treatment, in a few days, pain and hemorrhage ceased and the woman went about her ordinary household duties only, however, to bring about a return of her former condition. This state of affairs coutinued, sometimes worse and sometimes better, the contraction being trifling, and the hemorrhage merely a "show," until the afternoon of the 29th of October, when the pain became more severe, unaccompanied, however, by any flow. Upon examination *per vaginam*, the os was now found patulous and the state of affairs alarming, there being complete placenta praevia centralis, the placenta offering no resistance to the finger, but simply being lifted up by pressure applied from below. The attachment all the way around could be made out; also the point at which it had become separated for an inch or two; neither the head nor any portion of the foetus could be made out. The husband was advised of the situation, and notified of the dangers accompanying it, and as the pain had ceased upon my advent, I returned home, only a square from the house of my patient, leaving instructions to call me immediately upon the appearance of any flooding. A hasty summons came at ten o'clock that night, when hurrying to the bedside of my patient, I found the most profuse and alarming hemorrhage I have ever witnessed, blood pouring from the vagina in torrents, and with the utmost expedition on my part a large amount was lost, before thorough tamponing with absorbent cotton, through a Sims speculum, could be effected. This having been done, after an hour or two I returned home, the hemorrhage having been completely arrested. At 3 it returned slightly, the plugs were removed, tamponing again resorted to. The os being now more dilated than at my previous examination, I thought that operative procedures would soon become practicable, and requested that Dr. J. M. Lanham be called in consultation. He responded at once, but upon removing the plugs we decided that the amount of dilatations present was not sufficient for the operation of turning, and as that was the course we had determined to pursue, tamponing was again done ; violent contractions of the uterus succeeded, and in two or three hours, in spite of the plugs, the hemorrhage again became profuse. Upon examination it was found possible to insert the hand through the now dilatable os, and I proceeded at once to turn, Dr. Lanham administering chloroform. I introduced the right hand, passing the partially detached placenta on its left side, found the child high in the pelvis, the position dorso-anterior, right arm presenting. Passing over the head, the feet were seized and brought down, and, in view of the exhausted condition of the woman, delivery was concluded at once. The child was deeply cyanosed and did not breathe, but was taken charge of and resuscitated by Dr. Lanham. Considerable uterine atony was present at first, but several hypodermatic injections of the ergot, and vigorous bi-manual manipulation brought about sufficient contraction. For about ten days afterwards the patient was unable to lift her head from the pillow, being threatened with syncope at each attempt, but otherwise the recovery was uneventful. The child never throve and died upon the seventh day. My object in reporting the case is my conviction that turning was here most probably the only method of procedure which would have saved the woman's life. Due to the position of the child expectant measures, such as use of the tampon, no matter how thoroughly done, would have proved inefficient, and I believe that puncturing the membranes would have resulted disastrously had this been relied upon to stop the hemorrhage. The position of the child, I may add, was more than suspected, the abdominal walls being abnormally thin, so that palpation revealed the position more clearly than usual.

